# Misaligned Chromosomes are a Major Source of Chromosomal Instability in Breast Cancer

**DOI:** 10.1158/2767-9764.CRC-22-0302

**Published:** 2023-01-12

**Authors:** John B. Tucker, Sarah C. Bonema, Rebeca García-Varela, Ryan A. Denu, Yang Hu, Stephanie M. McGregor, Mark E. Burkard, Beth A. Weaver

**Affiliations:** 1 Cancer Biology Graduate Training Program, University of Wisconsin–Madison, Madison, Wisconsin.; 2 Molecular and Cellular Pharmacology Graduate Training Program, University of Wisconsin–Madison, Madison, Wisconsin.; 3 Department of Medicine, University of Wisconsin–Madison, Madison, Wisconsin.; 4 Medical Scientist Training Program, University of Wisconsin–Madison, Madison, Wisconsin.; 5 Department of Pathology and Laboratory Medicine, University of Wisconsin–Madison, Madison, Wisconsin.; 6 Department of Oncology/McArdle Laboratory for Cancer Research, University of Wisconsin–Madison, Madison, Wisconsin.; 7 Carbone Cancer Center, University of Wisconsin–Madison, Madison, Wisconsin.; 8 Department of Cell and Regenerative Biology, University of Wisconsin–Madison, Madison, Wisconsin.

## Abstract

Chromosomal instability (CIN), the persistent reshuffling of chromosomes during mitosis, is a hallmark of human cancers that contributes to tumor heterogeneity and has been implicated in driving metastasis and altering responses to therapy. Though multiple mechanisms can produce CIN, lagging chromosomes generated from abnormal merotelic attachments are the major cause of CIN in a variety of cell lines, and are expected to predominate in cancer. Here, we quantify CIN in breast cancer using a tumor microarray, matched primary and metastatic samples, and patient-derived organoids from primary breast cancer. Surprisingly, misaligned chromosomes are more common than lagging chromosomes and represent a major source of CIN in primary and metastatic tumors. This feature of breast cancers is conserved in a majority of breast cancer cell lines. Importantly, though a portion of misaligned chromosomes align before anaphase onset, the fraction that remain represents the largest source of CIN in these cells. Metastatic breast cancers exhibit higher rates of CIN than matched primary cancers, primarily due to increases in misaligned chromosomes. Whether CIN causes immune activation or evasion is controversial. We find that misaligned chromosomes result in immune-activating micronuclei substantially less frequently than lagging and bridge chromosomes and that breast cancers with greater frequencies of lagging chromosomes and chromosome bridges recruit more stromal tumor-infiltrating lymphocytes. These data indicate misaligned chromosomes represent a major mechanism of CIN in breast cancer and provide support for differential immunostimulatory effects of specific types of CIN.

**Significance:**

We surveyed the single-cell landscape of mitotic defects that generate CIN in primary and metastatic breast cancer and relevant models. Misaligned chromosomes predominate, and are less immunostimulatory than other chromosome segregation errors.

## Introduction

Chromosomal instability (CIN), the rate of persistent mitotic defects over consecutive divisions, results in random aneuploidy in daughter cells that is further shaped by selection (1, 2). Rates of CIN can vary widely from a single missegregated chromosome every few divisions to >10 chromosomes missegregated in a single division (3). Divergent rates of CIN have variable consequences. Low rates of CIN are well tolerated; animal models are uniformly viable and fertile and either display an incompletely penetrant oncogenic phenotype, often late in life, or have no discernable phenotype (4–8). High rates of CIN are lethal, likely due to loss of all copies of an essential chromosome (3, 9, 10). CIN+ cancer cell lines exhibit low rates of CIN (1–9 × 10^−3^ missegregations per chromosome), as measured by mitotic defects, which serve as a validated proxy for CIN (11, 12). Mathematical modeling confirms that this low CIN rate optimizes tumor suppressor loss and cellular heterogeneity while maintaining viability (1, 3, 13), likely because low CIN allows tumors to opportunistically select from a variety of karyotypes to increase fitness (14–18). Aneuploidy and CIN are associated with metastasis and poor prognosis in certain tumor types (19, 20); subdividing tumors based on rates of CIN reveals that high CIN tumors result in improved outcomes relative to those with lower rates (21–25). Consistent with this, CIN sensitizes breast cancer to the additional CIN induced by paclitaxel treatment (26). Despite the significance of CIN, its causes in cancer remain unknown.

Mitotic defects that produce CIN have been observed in tumors for over 100 years (27, 28). These defects include misaligned chromosomes that fail to align at the metaphase plate, chromosomes that lag behind the segregating masses of DNA late in mitosis, chromosomes that bridge the segregating DNA masses, and abnormal multipolar spindles that contain >2 spindle poles. Lagging chromosomes caused by inappropriate attachment of a single chromosome to both spindle poles (merotely) are the dominant cause of CIN in nontransformed (29–32) and cancerous (12, 33) cell lines. On the basis of this, it is currently expected that lagging chromosomes are the predominant cause of CIN in cancer (12, 34, 35). However, this has not been directly examined. Here we establish the landscape of mitotic defects in breast cancer.

One feature of mitotic defects is their potential to form micronuclei, in which isolated chromosomes are incorporated into a separate, small nuclear fragment. The nuclear envelopes of micronuclei are prone to rupture, which activates the cGAS/STING pathway responsible for surveilling the cytoplasm for double-stranded DNA (dsDNA) and initiates a proinflammatory immune response (36, 37). Micronuclei thus link CIN with immune activation, though emerging evidence suggests that certain inducers of micronuclei may be more immunostimulatory than others; micronuclei produced by Kif18 loss have more stable nuclear envelopes than those produced by nocodazole washout or radiation, though all induce lagging chromosomes (38). It remains unknown whether misaligned chromosomes are more or less immunostimulatory than lagging chromosomes or chromosome bridges in human cancer.

Here we directly quantify mitotic defects in primary and metastatic breast cancer. While multiple types of mitotic defects occur, misaligned chromosomes predominate and show the strongest correlation with CIN. Time-lapse analysis reveals that, despite the alignment of a fraction of misaligned chromosomes, those that remain misaligned at anaphase onset represent the major source of CIN. Misaligned chromosomes form micronuclei at a reduced frequency compared to lagging and bridge chromosomes, and are less immunogenic. These data demonstrate that misaligned chromosomes represent a major source of CIN in breast cancer and that the source of CIN influences the immune response.

## Materials and Methods

### Cell Culture

Cell lines were obtained from lab stocks and identities were confirmed via short tandem repeat testing. Cells tested *Mycoplasma* negative by fluorescence and/or ATP assay (Lonza LT07-418). Cal51 (RRID CVCL_1110) and MDA-MB-231 (RRID CVCL_0062) cells were cultured in DMEM + 10% FBS, 2 mmol/L l-glutamine, and 50 μg/mL pen/strep. MCF7 (RRID CVCL_0031) cells were cultured in the same media + 10 μg/mL insulin. MCF10A cells (RRID CVCL_0598) were cultured in DMEM:F12 with 1% horse serum, 20 ng/mL EGF, 500 μg/mL hydrocortisone, 100 ng/mL cholera toxin, 10 μg/mL insulin, and 50 μg/mL pen/strep. T47D cells (RRID CVCL_YX85) were cultured in RPMI1640 medium with 10% FBS, 2 mmol/L l-glutamine, and 50 μg/mL pen/strep. Each line was maintained at 37°C and 5% CO_2_.

### Patient Samples

Studies were conducted in accordance with recognized ethical guidelines as described in the U.S. Common Rule and with approval of the University of Wisconsin–Madison Institutional Review Board (IRB).

#### Tissue Microarray

The breast tissue microarray (TMA; refs. 39, 40) was compiled from primary breast tumor blocks from surgical specimens from patients with stage I–III breast cancer at the University of Wisconsin Carbone Cancer Center (protocol OS10111). The University of Wisconsin Health Sciences IRB approved the TMA creation and use of deidentified coded data (IRB approval no. 2010-0405), which was retrospectively collected. The IRB waived patient consent.

#### Organoids

Patient tissue was collected with informed consent from all patients in accordance with Health insurance Portability and Accountability Act (HIPAA) regulations, and all studies were approved by the IRB at the University of Wisconsin–Madison (IRB# UW14035, approval no. 2014-1053). Eligible patients were planned for ultrasound biopsy meeting certain criteria determined by the Diagnostic Radiologist. All subjects provided written informed consent. After performing the clinical biopsy, specimens were transferred into a prewarmed 37°C 24-well microplate. Tissue was washed with sterile PBS, and placed into a sterile petri dish. A total of 130 μL of primary mammary epithelium culture (PMEC) medium or Clever media was added. Tissue was then sliced with a sterile scalpel. A suspension of cellular material in media and thawed matrigel (1:1 ratio) was made and mixed. A droplet of 40 μL of this mixture was pipetted into the center of a well in the 24-well microplate. Gels were solidified in a 37°C incubator for 30 minutes and 500 μL of warm PMEC or Clever media was added to maintain three-dimensional cultures. Media was changed weekly.

#### Matched Primary and Metastatic Samples

A total of 18 cases of matched primary and metastatic breast tumor samples were retrospectively collected. Not all of the samples were used because of poor cellularity and/or sample quality. All studies were approved by the University of Wisconsin IRB (protocol UW16126 IRB, approval no. 2016-1379). The IRB waived patient consent.

### FISH

Two tissues sections per case were labeled with six centromeric probes for chromosomes 4, 10, and 17 and chromosomes 3, 7, and 9. For each chromosome, the percentage of cells with a nonmodal copy number was determined. CIN was calculated as the average percentage of cells that deviated from the modal number over the six chromosomes, as in refs. 26, 40.

### Microscopy

Mitotic defects were assessed in a subset of patients from the TMA as described in Supplementary Fig S1. Hematoxylin and eosin (H&E)-stained slides were observed using an Olympus BH-2 light microscope with a DPlan 40 0.65 NA objective. Slides from a separate deidentified cohort of 14 matched primary and metastatic cases were deparaffinized, rehydrated, and labeled with α-tubulin antibody (clone YL1/2 rat monoclonal IgG2a from Millipore Sigma MAB1864, RRID: AB_477579, 1:500 dilution) and secondary Alexa fluor 594 (1:200 dilution). DNA was stained using DAPI (1:1,000). Slides were analyzed using a Nikon Eclipse Ti-E inverted microscope with a 0.75 NA 40× objective.

For timelapse imaging, Cal51 and T47D cells with endogenously tagged H2B-mScarlet and enhanced green fluorescent protein (EGFP)-tubulin were seeded onto glass-bottom plates and maintained at 37°C and 5% CO_2_. A total of 20–24 hours prior to imaging, treated conditions received either 50 nmol/L GSK923235 or 50 nmol/L reversine immediately prior to imaging. Images were acquired every 5 minutes or every 2 minutes at 40× magnification on a Nikon Eclipse Ti -E inverted microscope in a Tokai Hit thermoregulating chamber. Images were acquired and compiled into montages using Nikon Elements software.

For immunofluorescence of fixed cells, cells were seeded on glass coverslips at low density in 12-well plates, allowed to grow until 50%–75% confluence and treated for 24 hours. Coverslips were preextracted for 5 minutes with 0.5% Triton X-100 in microtubule stabilizing buffer [MTSB: 100 mmol/L K-Pipes, pH 6.9, 30% (wt/vol) glycerol, 1 mmol/L Ethylene glycol-bis(2-aminoethylether)-*N,N,N′,N′*-tetraacetic acid (EGTA), 1 mmol/L MgSO_4_] and subsequently fixed in 4% formaldehyde in MTSB for 10 minutes at room temperature, washed once in PBS, and then blocked for 1 hour at room temperature in Triton Block. Primary antibodies were diluted in Triton Block and incubated overnight at 4°C. The following primary antibodies were used: cGAS D1D3G rabbit mAb #15102 Cell Signaling Technologies (RRID: AB_2799008; 1:200 dilution), α-tubulin clone YL1/2 rat monoclonal IgG2a Millipore Sigma MAB1864 (RRID: AB_477579; 1:2,000 dilution). After primary incubation, coverslips were washed 3 × 5 minutes in PBS+0.1% Triton X-100. Alexa Fluor (Invitrogen) secondary antibodies were diluted 1:200 in Triton Block. Coverslips were incubated in secondary antibodies for 45 minutes in the dark at room temperature and then washed three times in PBS+0.1%Triton X-100. Coverslips were counterstained with DAPI and mounted on glass slides with Vectashield mounting medium.

### 2′3′-cGAMP Measurements

A 2′3′-cGAMP ELISA Kit (Cayman Chemicals 501700) was used according to manufacturer's instructions. Lysates for each condition were prepared by mixing 100 μL of RIPA buffer (25 mmol/L Tris pH 7.4, 150 mmol/L NaCl, 1% nonidet P-40, 1% deoxycholic acid sodium salt) with previously collected flash frozen cell pellets consisting of 1 million cells stored at −80°C.

### Statistical Analysis

Statistical analysis was performed using PRISM version 9.0.0. Student *t* tests (two-tailed) were used to assess significance in comparative column analysis unless otherwise indicated in the figure legend. All single linear regressions and multiple linear regression slopes were significantly nonzero. Checks for adequacy of the regression models were performed through examination of q-q plots, plots of residuals versus fitted values, and testing residuals for normality (Shapiro–Wilk). No overt defects were found.

### Data Availability Statement

The data generated in this study are available upon request from the corresponding author.

## Results

### Misaligned Chromosomes are the Predominant Mitotic Error in Primary and Metastatic Breast Tumors

**FIGURE 1:**
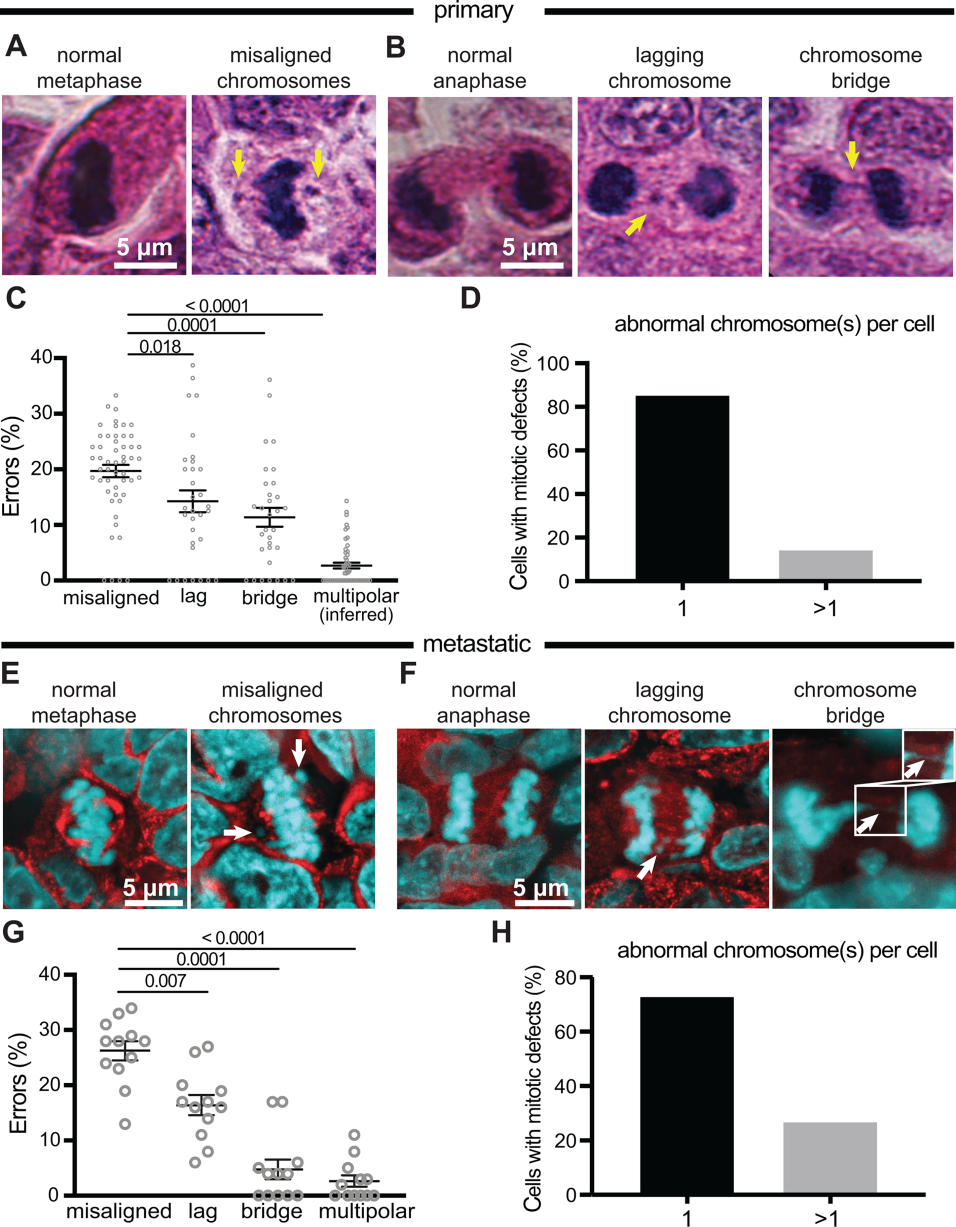
Misaligned chromosomes are the predominant mitotic error in primary and metastatic breast tumors. **A** and **B,** H&E images of normal and abnormal mitotic cells in primary breast cancer. Arrows indicate respective defects. **C,** Distribution of mitotic defects in primary breast tumors showing that misaligned chromosomes occur most frequently. *n* = 1,742 metaphase cells observed in 52 patients. *n* = 569 anaphase and telophase cells observed in 32 patients. **D,** Number of abnormal chromosomes in primary tumor cells with mitotic defects showing that, typically, only a single chromosome is affected. **E** and **F,** Immunofluorescence images of normal and abnormal mitotic cells in metastatic breast cancer. Arrows denote indicated defects. Inset in F shows that the DNA bridge is continuous. **G,** Distribution of mitotic defects found in 12 metastatic breast tumors showing that misaligned chromosomes are the most common defect. *n* = 52 metaphase and 20 anaphase and telophase cells on average per case. **H,** Number of abnormal chromosomes in metastatic cancer cells with mitotic defects showing that in the majority of divisions, only a single chromosome is affected. *P* values derived from unpaired *t* tests.

To survey the landscape of mitotic defects that contribute to CIN in breast tumors, we analyzed the subset of sufficiently proliferative primary breast cancers from a breast tissue microarray [Supplementary Fig. S1 (40)]. A total of 62 cancer cases with a Ki67 (proliferation) score of ≥20%, as well as seven normal cases, were analyzed. These cases included stage I–III cancers of all major subtypes (Supplementary Table S1). Mitotic figures previously associated with chromosome missegregation including lagging chromosomes, chromosome bridges, and misaligned chromosomes in cells with a well-established metaphase plate were quantified on H&E-stained tumor sections. Multiple types of mitotic figures consistent with chromosome missegregation were observed, and their frequency was similar among subtypes (Supplementary Fig. S2A and S2B). In contrast to our expectation that lagging chromosomes would predominate, misaligned chromosomes were the most common mitotic phenotype observed, followed by lagging chromosomes and chromosome bridges (Fig. 1A–C).

Because H&E staining precludes spindle pole demarcation, multipolarity was inferred on the basis of “Y” and “X” shaped metaphase plates and groupings of ≥2 chromatin masses later in mitosis. Inferred multipolar spindles were the least common mitotic defect (Fig. 1C), though we note that this indirect measure could provide an underestimate. Highly aberrant mitotic cells in which multiple chromosomes were missegregated were rare; in approximately 85% of cells with misaligned, lagging or bridge chromosomes, only a single chromosome was affected (Fig. 1D). Thus, misalignment of individual chromosomes represents the most common mitotic phenotype associated with CIN in primary breast cancer.

We next analyzed metastatic breast cancer to establish the mitotic defects that contribute to CIN. A separate cohort of 12 matched primary and metastatic tumors was analyzed by immunofluorescence (Fig. 1E and F; Supplementary Table S2). The pattern of mitotic phenotypes in metastatic breast cancer mirrored that in primary breast cancer, though misaligned chromosomes were somewhat more common in metastatic samples (Fig. 1C vs. Fig. 1G). Similar to primary breast cancer, misaligned chromosomes predominated in metastatic breast tumors, followed by lagging chromosomes, chromosome bridges, and multipolar spindles (Fig. 1G). As in primary cancer, most abnormal divisions involved only a single chromosome (Fig. 1H). Lagging chromosomes can originate from merotelic attachments or from premitotic errors that generate acentric chromatin fragments (41). Anti-centromere antibody staining in eight primary cancers revealed that approximately two-thirds of lagging chromosomes in anaphase contained centromeres, consistent with a previous report that mitotic errors, not S phase–derived fragments, represent the predominant cause of lagging chromosomes (ref. 35; Supplementary Fig. S3). These results demonstrate that multiple types of CIN-inducing mitotic defects occur in primary and metastatic breast tumors, with misaligned chromosomes predominating.

### CIN Increases as Tumors Progress, Predominantly Due to an Increase in Misaligned Chromosomes

**FIGURE 2:**
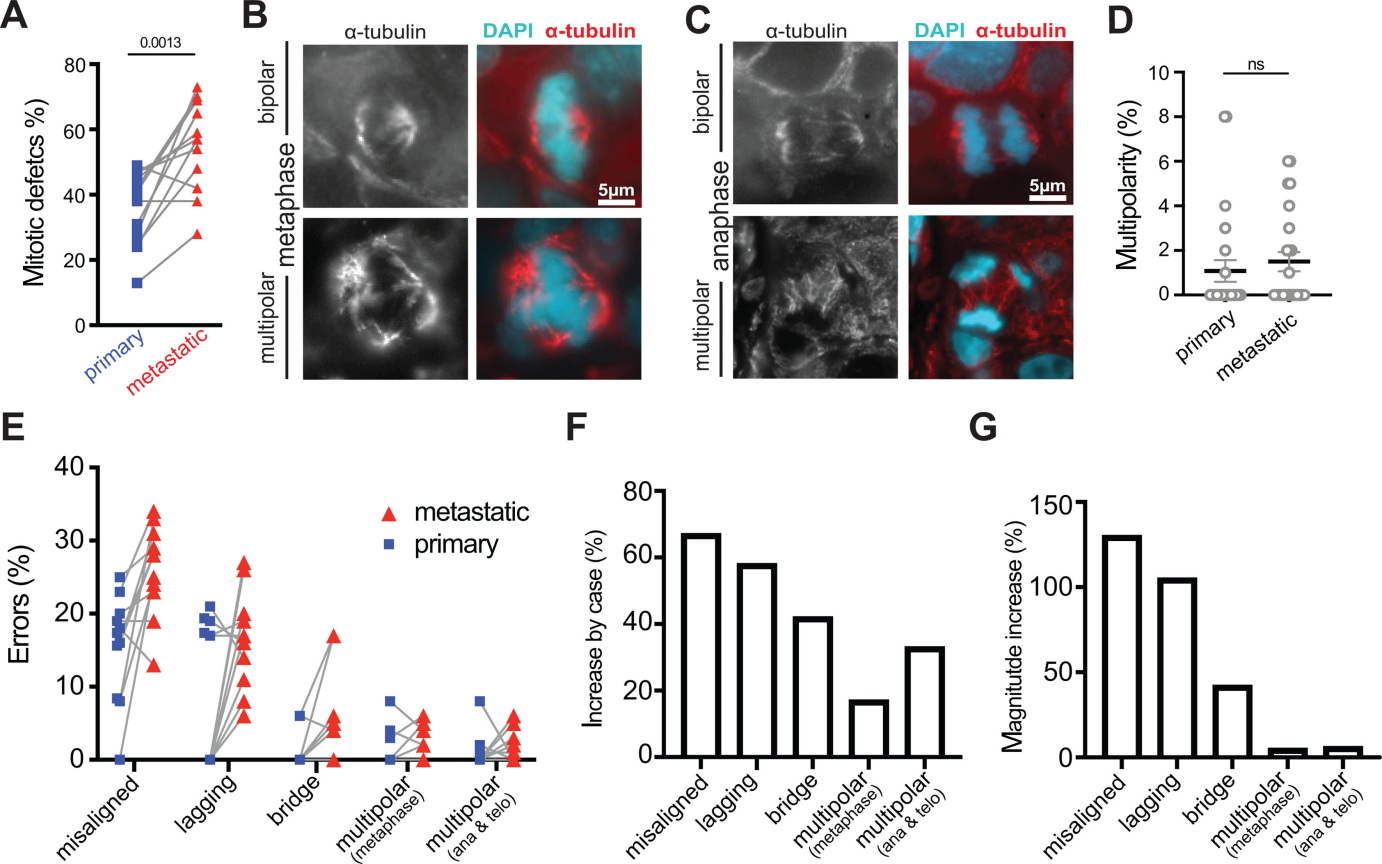
CIN increases as tumors progress, predominantly due to an increase in misaligned chromosomes. **A,** Comparison of total mitotic defects in paired primary and metastatic breast cancer patient samples. Lines connect paired primary and metastatic tumor samples. **B,** Bipolar (top) and multipolar (bottom) metaphase spindles. **C,** Bipolar (top) and multipolar (bottom) anaphase spindles. **D,** Quantification of spindle multipolarity in 12 primary and 12 metastatic breast cancer samples showing a range of low levels of multipolarity in both. *P* = 0.0527 derived from unpaired *t* test. **E**–**G,** Quantification of mitotic defects in matched primary and metastatic breast tumors. *n* = 364 and 628 metaphase cells observed in 12 primary and metastatic patient samples, respectively. *n* = 178 and 239 anaphase and telophase cells observed in 12 primary and metastatic patient samples, respectively. **E,** Incidence of multipolar spindles and misaligned, lagging, and bridge chromosomes in matched primary and metastatic breast cancers. Matched cases are connected by gray lines. **F,** The percentage of cases in which the indicated defect increased by ≥1 SD in the metastatic as compared with the primary site. **G,** The magnitude of the observed change in each defect summed across all cases. ns = not significant.

Metastatic cancers have previously been reported to have higher levels of genomic instability than unmatched primary tumors based on bulk analysis of tumor cells (42, 43). To determine whether CIN increases as individual tumors metastasize, and the associated mitotic abnormalities, 12 matched primary and metastatic breast cancers were assessed for mitotic defects. Indeed, mitotic errors that cause CIN increased in 10 of 12 cases as they progressed from primary to metastatic cancer (Fig. 2A). Only one case showed a decrease in CIN as it metastasized, which was due to a reduction in the incidence of lagging chromosomes. Immunofluorescence measurement of spindle multipolarity in primary tumors was in good agreement with inferred multipolarity based on H&E staining (1.5% ± 0.5% for immunofluorescence vs. 1.1% ± 0.3% for H&E; Figs. 1C and 2B–D). Spindle multipolarity was similar in primary and metastatic tumors (Fig. 2D). In contrast, substantial increases in misaligned, lagging, and bridge chromosomes occurred as matched tumors progressed (Fig. 2E–G). Missegregation of individual chromosomes occurred far more often than missegregation of large numbers of chromosomes on multiple spindle poles (Fig. 2E). The incidence of misaligned and lagging chromosomes rose in most tumors as they progressed to become metastatic (Fig. 2E and F). Increases in chromosome bridges were also common, as were gains in multipolar spindles, particularly in late stages of mitosis (Fig. 2F). However, the magnitude of the increase was greatest for misaligned chromosomes, followed by lagging chromosomes and chromosome bridges, with multipolar spindles showing only a minor change (Fig. 2G). These data support the conclusion that metastatic cancers exhibit higher rates of CIN than primary cancers. Furthermore, they suggest that misaligned chromosomes, followed by lagging chromosomes, make the largest contribution to the increased rate of CIN in metastatic breast cancer.

### Misaligned Chromosomes Correlate with CIN in Primary Breast Cancer

**FIGURE 3:**
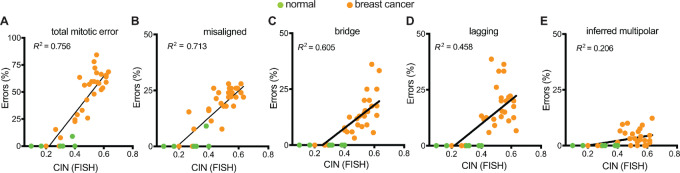
Misaligned chromosomes correlate with CIN in primary breast cancer. Correlation of CIN [based on variation in copy number of six chromosomes measured by interphase FISH (Methods (40)] using simple linear regressions with all types of mitotic errors (**A**), misaligned chromosomes (**B**), chromosome bridges (**C**), lagging chromosomes (**D**), and inferred multipolarity (**E**) demonstrating that misaligned chromosomes have the strongest correlation with CIN. *n* = 1,204 metaphase cells observed in 38 patients. *n* = 567 anaphase and telophase cells observed in 38 patients. *n* = 60 mitotic cells observed in normal tissue. **A**–**D,***P* < 0.0001; **E**, *P* = 0.0042.

Mitosis is a robust, dynamic process and cells have evolved numerous mechanisms for correcting potential mitotic errors, including mechanisms to prevent segregation of misaligned chromosomes, facilitate proper segregation of lagging chromosomes, and focus multipolar spindles (31, 32, 44, 45). As a proxy for determining which mitotic errors persist and contribute to CIN in primary breast cancer, we correlated the observed mitotic defects with CIN, as assessed by the intercellular variability in six chromosomes scored using interphase FISH (40). CIN was quantified by averaging the percentage of cells with a nonmodal copy number of each chromosome [Methods (26, 40)]. As expected, mitotic defects positively correlated with CIN when considered together (Fig. 3A). Analysis of specific mitotic defects revealed misaligned chromosomes showed the strongest correlation with CIN (Fig. 3B), followed by chromosome bridges (Fig. 3C), and lagging chromosomes (Fig. 3D). Inferred multipolar spindles did not correlate with CIN (Fig. 3E), likely due to their low frequency and likelihood of generating inviable progeny. Multiple linear regression analysis of all defects revealed that misaligned and bridge chromosomes significantly contributed to the association of CIN with mitotic defects, while lagging chromosomes and multipolar spindles did not (Supplementary Table S3). These data demonstrate the strong positive correlation between misaligned chromosomes and CIN in primary breast cancer.

### Misaligned Chromosomes Result in Fewer Micronuclei and Less Immune Activation than Other Mitotic Defects

**FIGURE 4:**
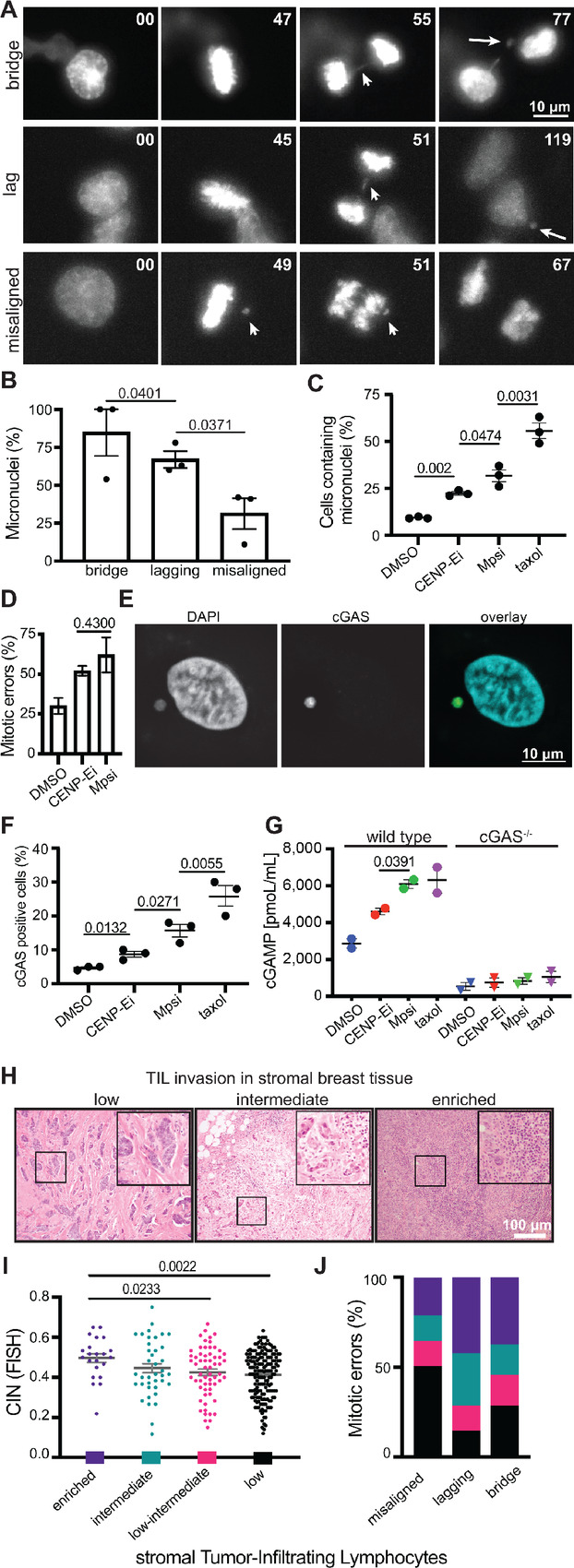
Misaligned chromosomes result in fewer micronuclei and less immune activation than other mitotic defects. Still images from 24-hour timelapse analysis at 2-minute intervals of Cal51 cells with endogenously labeled histone-H2B and EGFP-α-tubulin showing the most common fate of chromosome bridges (top, arrow), lagging chromosomes (middle, arrow), and misaligned chromosomes (bottom, arrows). Long arrows in far right panels indicate micronuclei. **B,** Frequency of micronucleus formation in daughter cells following each mitotic defect. **C,** Cells containing micronuclei after chemical induction of misaligned chromosomes with 50 nmol/L of the CENP-E inhibitor GSK923296, or lagging or bridge chromosomes due to 50 nmol/L of the Mps1 inhibitor reversine. A total of 10 nmol/L taxol was used as a positive control to generate micronucleated and multinucleated daughter cells. **B** and **C,***n* > 125 cells per condition in each of three independent replicates. **D,** Frequency of mitotic defects induced by GSK (misaligned chromosomes that persist until anaphase onset) and reversine (lagging and bridge chromosomes). *n* = 15–20 mitotic cells per condition in each of three independent movies. **E,** Image of cGAS positive micronucleus. **F,** cGAS positive cells 48 hours after indicated treatment. *n* >125 cells in each of three independent replicates. Taxol was used as a positive control (51). **G,** ELISA quantification of 2′3′-cGAMP in cells following 48-hour treatment in parental wild type and CRISPR edited cGAS knockout MDA-MB-231 cells. **H,** Representative images of stromal TIL infiltration. **I,** CIN positively correlates with sTIL enrichment. ANOVA *P* value = 0.0154. sTIL scores: low <10%, low-intermediate 10%–20%, intermediate 20%–40%, enriched >40%. **J,** Frequency of sTIL infiltration categories in tumors delineated by mitotic defect. Tumors with misaligned chromosomes most frequently have low sTILs while tumors with lagging and bridge chromosomes are most frequently enriched. The specified mitotic errors occur in 0%–20% of tumor cells. *P* < 0.001, *χ*^2^ test. Other *P* values derived from unpaired *t* test.

It is well established that chromosomes separated from the segregating DNA masses can be encapsulated into micronuclei (12, 46), but the relative rates at which misaligned, lagging, and bridge chromosomes form micronuclei remained unknown. To test this, we treated Cal51 triple-negative (negative for estrogen receptor, progesterone receptor, and HER2 amplification) breast cancer cells with either GSK923235 to induce misaligned chromosomes by inhibiting the plus end directed kinesin motor CENP-E or with reversine, an Mps1 inhibitor that accelerates mitotic transit to generate lagging and bridge chromosomes (47). Timelapse analysis was used to track Cal51 cells with endogenously labeled histone H2B-mScarlet and EGFP-tubulin, to label chromosomes and microtubules, respectively, at 2-minute intervals over 24 hours. Misaligned, lagging, and bridge chromosomes were followed to determine the frequency with which they formed micronuclei (Fig. 4A). Chromosome bridges and lagging chromosomes typically resulted in micronuclei (Fig. 4B). Misaligned chromosomes formed micronuclei with substantially less frequency (Fig. 4B). Consistent with this, substantially more cells developed micronuclei after inhibition of Mps1 than CENP-E (Fig. 4C), even though misaligned chromosomes that persisted until anaphase onset occurred as frequently in CENP-E inhibited cells as lagging and bridge chromosomes in Mps1 inhibited cells (Fig. 4D).

Micronuclei have been shown to generate an immune response via the cyclic GMP‐AMP synthase (cGAS)/stimulator of interferon genes (STING) pathway (36, 48). cGAS binds dsDNA in micronuclei due to leaky nuclear envelopes. Consistent with misaligned and lagging chromosomes resulting in higher levels of micronuclei than misaligned chromosomes, Mps1 inhibition produced higher rates of cGAS positive cells than CENP-E inhibition (Fig. 4E and F). cGAS activation by dsDNA triggers synthesis of the secondary messenger 2′3′-cyclic guanosine monophosphate–adenosine monophosphate (cGAMP), which binds STING to activate an immune response (49, 50). Consistent with lagging and bridge chromosomes eliciting a heightened cGAS response relative to misaligned chromosomes, Mps1 inhibited cells showed higher levels of cGAMP than CENP-E inhibited cells (Fig. 4G, left). Isogenic cells in which CRISPR/Cas9 had been used to knock out cGAS (51) showed minimal cGAMP, confirming the specificity of the cGAMP response (Fig. 4G, right).

Experimental induction of CIN has been reported to induce an immune response (52, 53), though sequencing of human tumors has associated CIN with immune evasion (54, 55). To determine whether CIN positively or negatively correlated with immune infiltration in breast cancer, we assessed 58 primary tumor samples in our breast tissue microarray for stromal tumor-infiltrating lymphocytes (sTIL) and correlated with CIN as assessed by six chromosome FISH. Interestingly, higher CIN tumors were significantly enriched for sTILs (Fig. 4H and I). This is apparently driven by lagging chromosomes and chromosome bridges, as these defects were primarily associated with high sTIL enrichment while misaligned chromosomes associated with low sTIL infiltration (Fig. 4J). These results suggest that specific mitotic defects can have differential postmitotic effects on the innate immune response, and link CIN caused by lagging and bridge chromosomes to immune activation.

### Misaligned Chromosomes Persist at Anaphase Onset and Provide the Primary Cause of CIN in Breast Cancer Cells

**FIGURE 5:**
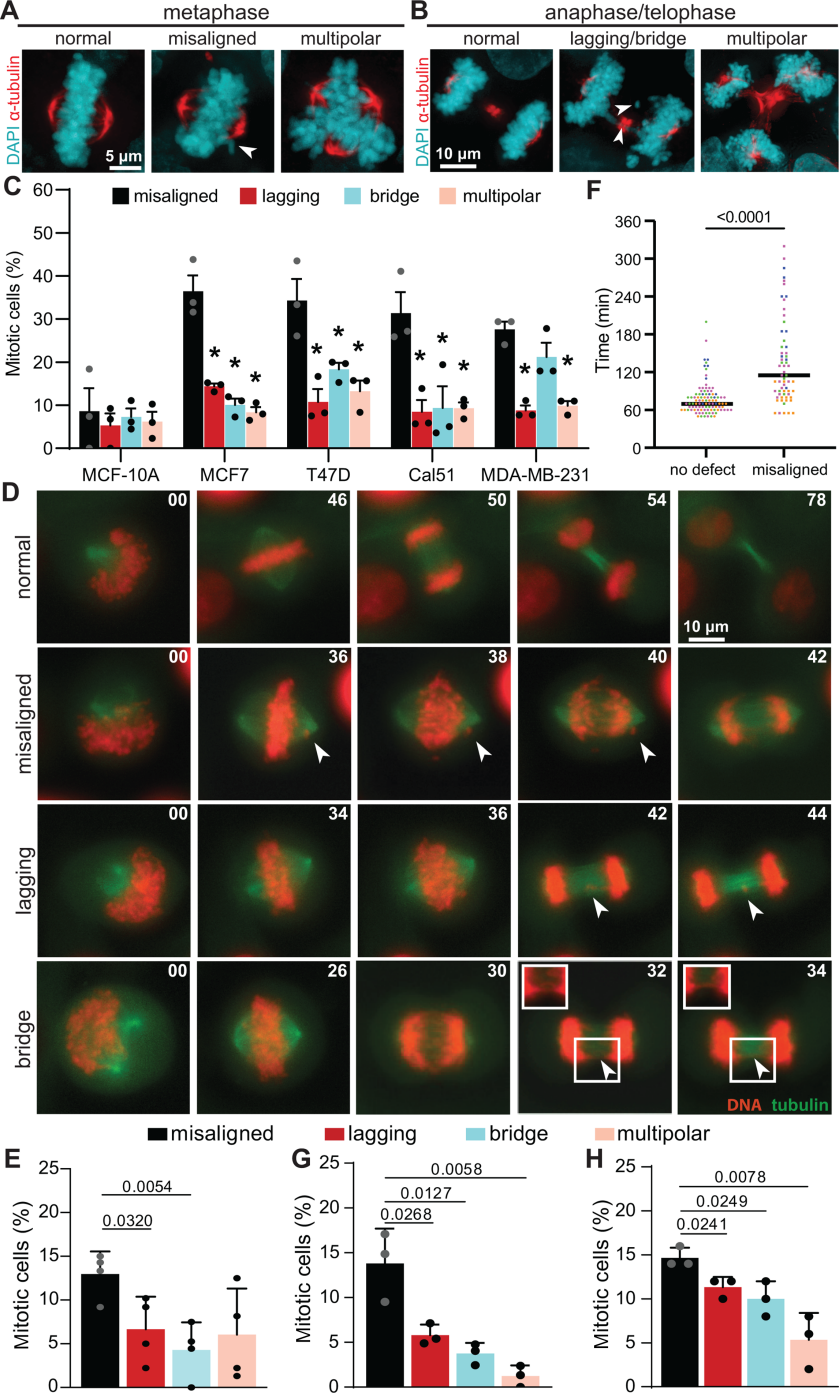
Misaligned chromosomes that persist until anaphase onset are a major cause of CIN in breast cancer. **A** and **B,** Immunofluorescence images of T47D cells. Arrowheads denote indicated defects. **C,** Frequencies of mitotic defects (mean ± SD) in nontransformed (MCF10A) and breast cancer cell lines showing that metaphase cells with a well-defined metaphase plate and one or more misaligned chromosomes that are clearly separated from the plate predominate. *n* > 150 metaphase cells and 150 anaphase+telophase cells in three biological replicates. *, *P* < 0.05, unpaired *t* test. **D,** Still images from timelapse analysis of mitotic defects in T47D cells with endogenously labeled histone H2B and α-tubulin, to label DNA and microtubules, respectively, showing normal division (top; Supplementary Movie S1), a misaligned chromosome that persists through anaphase onset (row 2; Supplementary Movie S2), a lagging chromosome (row 3; Supplementary Movie S3), and a chromosome bridge (bottom; Supplementary Movie S4). **E,** Quantification of mitotic defects in Cal51 cells with endogenously labeled histone H2B and α-tubulin imaged at 5-minute intervals. *n* = 40–73 mitotic cells in each of four biological replicates. **F,** Duration of mitosis (NEB to daughter cell flattening) in Cal51 cells with and without misaligned chromosomes in **E**, showing cells with misaligned chromosomes in metaphase exhibit a substantial mitotic delay. **G** and **H,** Quantification of mitotic defects observed using timelapse microscopy in endogenously tagged Cal51 (**G**) or T47D (**H**) cells with 2-minute acquisition intervals. *n* = 41–74 cells in each of three biological replicates. *P* values derived from unpaired *t* tests.

To directly assess whether mitotic errors, including misaligned chromosomes, correct during division, we acquired patient-derived organoids (PDO) from primary breast cancers. Similar to primary and metastatic breast cancer, misaligned chromosomes were the most common defect identified in PDOs, followed by lagging and bridge chromosomes, while multipolar spindles were the least frequent (Supplementary Fig. S4). Despite extensive efforts, we were unable to use timelapse analysis to image a sufficient number of mitotic cells in PDOs for quantitative analysis. We therefore turned to breast cancer cell lines. Quantification of estrogen receptor–positive (MCF7 and T47D) and triple-negative (Cal51 and MDA-MB-231) breast cancer cell lines as well as nontransformed MCF10A breast cells revealed that, as in primary and metastatic breast cancer, chromosome alignment defects in cells with a clearly established metaphase plate were the most common mitotic defect in the majority of breast cancer cell lines assessed (Fig. 5A–C).

To test whether misaligned chromosomes persisted until anaphase onset, resulting in CIN, we performed timelapse analysis of Cal51 cells endogenously tagged with EGFP-α tubulin and histone H2B-mScarlet (Fig. 5D). Though a fraction of misaligned chromosomes aligned prior to anaphase onset, the portion of chromosomes that remained misaligned at anaphase onset were significantly more frequent than other mitotic defects (Fig. 5E). Misaligned chromosomes typically have at least one unattached kinetochore, which activates the mitotic spindle assembly checkpoint and delays mitosis (56, 57). Consistent with this, metaphase cells with misaligned chromosomes showed a marked mitotic delay (Fig. 5F), though they ultimately entered anaphase and proceeded through mitosis.

We considered the possibility that the misaligned chromosomes had aligned during the 5-minute imaging interval. We therefore imaged endogenously labeled Cal51 and T47D cells at 2-minute intervals. In both cell lines, chromosomes that remained misaligned at anaphase onset were the most common mitotic defect observed (Fig. 5G and H). Though these misaligned chromosomes were initially detectable in anaphase, a large majority of them rapidly merged with the main masses of segregating DNA in anaphase A, becoming undetectable. Only a small percentage remained distinct from the segregating DNA masses in anaphase B (Supplementary Fig. S5A and S5B). Because anaphase A is quite brief, 2.4–3.7 minutes, representing only 6%–7% of the total time cells spend in anaphase and telophase (Supplementary Fig. S5C and S5D), analysis of fixed cells is likely to dramatically underestimate the frequency with which misaligned chromosomes persist until anaphase onset. Because misaligned chromosomes at anaphase onset are the most common mitotic defect observed, and lagging chromosomes are regularly segregated to the appropriate daughter cell (32, 44, 58) while misaligned chromosomes at anaphase onset necessarily result in chromosome missegregation, we conclude that chromosomes that remain misaligned at anaphase onset provide the primary source of CIN in these cells. Together, these data reveal a consistent landscape of mitotic defects in primary and metastatic breast cancer as well as breast cancer PDOs and cell lines, in which multiple mitotic defects contribute to CIN, with misaligned chromosomes predominating, followed by lagging and bridge chromosomes, and multipolar divisions.

## Discussion

CIN is a common feature of cancers that can affect metastasis and drug resistance, is induced by the existing therapies paclitaxel and radiation, and is the focus of novel anticancer strategies. A clear understanding of the underlying CIN in cancer is therefore critical for optimizing existing and emerging treatment options. Previous experiments convincingly demonstrated that lagging chromosomes were the predominant source of CIN in nontransformed cells and a small subset of CIN cancer cell lines, which did not exhibit misaligned chromosomes at anaphase onset. Generalization of these findings resulted in the prevailing view that lagging chromosomes are the primary cause of CIN in cancer and that misaligned chromosomes align prior to anaphase and therefore do not contribute to CIN. However, this conclusion was based on examination of a relatively small number of CIN cancer cell lines and did not include examination of patient tumors. Here we quantify mitotic defects in primary and metastatic breast cancer and discover that, while lagging chromosomes are indeed common, misaligned chromosomes are even more prevalent. Cells have mechanisms for correcting both misaligned and lagging chromosomes to ensure that they are segregated into the correct daughter cell. It had previously been thought that misaligned chromosomes are reliably aligned before cells enter anaphase, and are therefore not missegregated. However, here we show that in breast cancer cell lines chromosomes that remain misaligned at anaphase onset are the most common mitotic defect. While this article was in review, a second group reported that other cancer cell lines commonly enter anaphase in the presence of misaligned chromosomes (59), providing independent validation that misaligned chromosomes are a source of CIN. Cells have at least two mechanisms to ensure that lagging chromosomes are segregated into the appropriate daughter cell and therefore do not contribute to CIN (32, 44, 58). Lagging chromosomes, some of which are expected to be correctly segregated, are less common than misaligned chromosomes that persist until anaphase onset—which are necessarily missegregated—in breast cancer models, emphasizing the importance of misaligned chromosomes in inducing CIN. In primary breast cancer, both misaligned chromosomes and chromosome bridges showed stronger correlation with CIN than lagging chromosomes. These data demonstrate that, while multiple types of mitotic errors contribute, misaligned chromosomes provide a major source of CIN in breast cancer.

A recent study found that misaligned chromosomes, particularly those on the far side of the spindle pole, tend to become ensheathed in endomembranes [remnants of the endoplasmic reticulum (ER), nuclear envelope, and Golgi apparatus that become dispersed during mitosis and are densely packed in the cell periphery (60)]. A total of 39% of ensheathed misaligned chromosomes formed micronuclei, while misaligned chromosomes not completely surrounded by endomembranes (“free” misaligned chromosomes) did not. The rate of micronuclei formation from misaligned chromosomes we observed (31.3%) suggests that most of the misaligned chromosomes we observed were ensheathed.

Classic studies that analyzed a small subset of CIN cancer cell lines identified lagging chromosomes but not misaligned chromosomes at anaphase onset (12, 33). Coupled with the ability of these cells to delay transit through mitosis in the presence of high doses of microtubule poisons, the absence of misaligned chromosomes that were ultimately missegregated was interpreted as evidence that cancer cell lines have an active mitotic spindle assembly checkpoint (33, 61). However, it is now clear that the mitotic checkpoint acts as a rheostat rather than an on-off switch (62–64). The strength of the mitotic checkpoint signal varies in response to the number of unattached kinetochores generating a mitotic checkpoint signal and the levels of mitotic checkpoint proteins (62–64), which are commonly upregulated and downregulated in cancer (8). The ability to extend mitosis indicates that the mitotic checkpoint is functional but not necessarily that the checkpoint is sufficiently robust to perform its biological function of delaying anaphase in the presence of a single unattached kinetochore to prevent CIN. In cells that enter anaphase with misaligned chromosomes after a mitotic delay due to reduction of CENP-E (59, 65, 66), Mad2-GFP is no longer detected at the kinetochores of misaligned chromosomes by the time the cells enter anaphase and Mad1 is virtually undetectable at kinetochores in early anaphase (59) and in subsequent micronuclei (65). These results are consistent with satisfaction of mitotic checkpoint signaling, though they do not explain why the checkpoint is improperly silenced in the presence of chromosomes that are ultimately missegregated. However, reduction of CENP-E reduces the strength of the mitotic checkpoint by decreasing the amount of Mad1, Mad2, and BubR1 that are recruited to unattached kinetochores (67). Thus, these results are also consistent with the mitotic checkpoint being intact but of insufficient strength to prevent chromosome missegregation (60). Similarly, here we observed that breast cancer cells with misaligned chromosomes delay in mitosis, consistent with a mitotic checkpoint response, but ultimately proceed through mitosis and missegregate the misaligned chromosome. An interesting topic for future work will be to determine whether the checkpoint is inappropriately silenced in these cells or whether the checkpoint is of insufficient strength to prevent CIN.

Though chromosomes that remained misaligned at anaphase onset were not observed in a small subset of CIN cancer cell lines (12, 33), they have been observed in more recent experiments in colorectal cancer organoids and freshly generated ovarian cancer cell lines (68, 69). Several ovarian cancer cell lines were capable of delaying or arresting in mitosis in response to microtubule poisons, though this was not tested in lines that entered anaphase with misaligned chromosomes (68). Interestingly, lagging and/or bridge chromosomes occurred more commonly than misaligned chromosomes in both colorectal and ovarian contexts. It remains to be determined whether this reflects culture-induced variation or, more likely, the underlying biology of distinct cancer types.

These data have implications for cancer evolution. Missegregation of single misaligned, lagging, or bridge chromosomes occurs much more commonly than the massive chromosome missegregation that occurs from a multipolar division. Multipolar divisions cause large-scale genomic rearrangements and, though they typically produce inviable daughter cells (26, 45), are considered examples of saltatory genome evolution that produce “hopeful monsters” (70). Mitotic defects involving single chromosomes positively correlated with CIN in primary breast cancers, but multipolar spindles did not. As tumors progressed to become metastatic, the incidence of single chromosome missegregation increased substantially, while multipolar spindles—particularly those that were maintained into anaphase and telophase, which is necessary for them to induce high rates of CIN—remained quite infrequent. Since saltatory evolution typically produces inviable “maladapted monsters” and must occur with substantial frequency to produce viable “hopeful monsters,” these data suggest a gradual, continuous evolution of these breast tumors rather than punctuated evolution.

Lagging chromosomes caused by distinct insults were previously shown to differ in their propensity to have unstable nuclear envelopes that recruit cGAS (38). Here we show that lagging and bridge chromosomes are more potent than misaligned chromosomes in inducing micronuclei. A recent publication came to an opposing conclusion (59). The likely reason for this is that they only considered cells that “exit mitosis” with misaligned chromosomes—rather than those that enter anaphase with misaligned chromosomes—when calculating the frequency with which misaligned chromosomes produce micronuclei. As most misaligned chromosomes join with the main masses of segregating DNA in anaphase A, those that remain misaligned at mitotic exit are the small subset of misaligned chromosomes that are most likely to form micronuclei. Our balanced analysis considers all misaligned, lagging, and bridge chromosomes that occur after anaphase onset equally, and finds that misaligned chromosomes are substantially less likely than lagging chromosomes or chromosome bridges to result in micronuclei. Unstable nuclear envelopes occurred with similar frequency in micronuclei generated by misaligned, lagging or bridge chromosomes, but because lagging and bridge chromosomes result in micronuclei more commonly than misaligned chromosomes, the overall production of cGAMP is higher in response to these defects. In primary breast cancers, sTIL infiltration increased with CIN. Lagging and bridge chromosomes appeared particularly adept at recruiting sTILs, with misaligned chromosomes less so, though the co-occurrence of all three mitotic defects to various extents in each tumor confounds this analysis somewhat. Overall, these data suggest that misaligned chromosomes are less immunogenic than lagging and bridge chromosomes, offering a potential explanation for CIN breast cancers that are immunologically “cold.”

Previous measurements of CIN in breast cancer report that approximately 50% of breast cancers exhibit mitotic defects that cause CIN (14, 40, 71). Here we show that in a large majority of cases, only a single chromosome is missegregated, resulting in a CIN rate in diploid tumors of 5.7 × 10^−3^ missegregations per chromosome. This rate is consistent with the range previously observed in cancer cell lines [1–9 × 10^−3^ missegregations per chromosome (11, 12)], which mathematical modeling suggests maximizes viability and heterogeneity (1, 3, 13). We predict that this low, tolerable rate of CIN sensitizes these breast cancers to treatments that further increase CIN over a maximally tolerated threshold. In this case, the half of breast cancers that respond to taxanes are the same cancers with these low, tolerable levels of mitotic defects prior to treatment, while resistant tumors lack CIN. Thus, pretreatment CIN could be used as a biomarker of taxane response. Indeed, tumors with preexisting CIN were more sensitive to the additional CIN induced by taxane in metastatic breast cancer (26). Combining CENP-E inhibition with paclitaxel increased CIN due to misaligned chromosomes and substantially reduced daughter cell viability in breast cancer cell lines, further supporting mitosis as a viable target for anticancer treatment (26). Though single misaligned chromosomes are common in breast cancer, increasing their rate could represent a technically accessible method to sensitize the approximately 50% of tumors resistant to taxane treatment.

## Supplementary Material

Movie S1 Normal division

Movie S2 Misaligned chromosome that persists through anaphase onset

Movie S3 Lagging chromosome

Movie S4 Chromosome bridge

Fig FS1 Figure S1. Pipeline to assess CIN in primary breast cancer. A. A tissue microarray containing 377 breast cancer and 17 normal breast samples was evaluated for proliferation by Ki67 and CIN based on 6 chromosome FISH (39). H&E stained sections for cases with sufficient proliferation to assess mitotic cells with a range of CIN scores were analyzed for mitotic defects. Linear correlations were used to compare CIN by FISH to mitotic defects in tumor sections.

Fig FS2 Figure S2. Breast tumor subtypes show similar rates of mitotic defects. A. Triple Negative (TN) and HER2+ tumors show higher CIN as assessed by 6-chromosome interphase FISH (see Methods) than hormone (ER and/or PR) receptor positive (HR+) tumors. n = 235, 45, 46 respectively. B. Rates of mitotic defects are similar in all subtypes.

Fig FS3 Figure S3. Lagging chromosomes in breast cancer are primarily mitotic in origin. A-B. Images of lagging chromosomes lacking a centromere, which are likely to occur due to premitotic defects (A) or containing their centromere, which are likely to arise due to mitotic defects (B). C. Quantification of lagging chromosomes in 8 primary tumors showing acentric (ACA-) fragments are less frequent than lagging chromosomes containing centromeres. Data represent mean +/- SD.

Fig FS4 Figure S4. Misaligned chromosomes are the predominant mitotic error in breast cancer organoids. A. Biopsies of primary breast cancer were grown in Matrigel suspension into patient-derived organoids (PDOs), which were prepared for immunofluorescence and confocal imaging. B. Representative images of mitotic cells with misaligned chromosomes observed in PDOs. C. Frequency of mitotic defects assessed in PDOs from 10 patients. P values derived from unpaired t-test.

Fig FS5 Figure S5. Chromosomes that are misaligned at anaphase onset are rapidly incorporated into the main DNA masses during anaphase A. A-B. Timelapse analysis of T47D (A) and Cal51 (B) breast cancer cells with endogenously labeled histone H2B and a-tubulin showing the fate of misaligned chromosomes after anaphase entry. In cells that entered anaphase with misaligned chromosomes, the majority of the misaligned chromosomes reincorporated into the main masses of segregating DNA during anaphase A (left columns). A smaller portion of misaligned chromosomes remained distinct from the segregating DNA masses and therefore visible throughout anaphase A (middle column). A small minority of misaligned chromosomes were detectable for at least 2 minutes during anaphase B (right column). n>25 cells with misaligned chromosomes in 3 biological replicates. C-D timing of anaphase and telophase in T47D (C) and Cal51 (D) cells. n>55 cells in 3 biological replicates.

Table TS1 Table S1. Patient characteristics

Table TS2 Table S2. Patient characteristics of matched primary and metastatic cases

Table TS3 Table S3. Results from multivariable regression, related to Figure 3.
