# Supplementary material for: Misaligned Chromosomes are a Major Source of Chromosomal Instability in Breast Cancer
Source: Cancer Res Commun. 2023 Jan 12;3(1):54–65. doi: 10.1158/2767-9764.CRC-22-0302 (PMC10035514; doi:10.1158/2767-9764.CRC-22-0302)
Supplement: Fig FS1 — Figure S1. Pipeline to assess CIN in primary breast cancer. A. A tissue microarray containing 377 breast cancer and 17 normal breast samples was evaluated for proliferation by Ki67 and CIN based on 6 chromosome FISH (39). H&E stained sections for cases with sufficient proliferation to assess mitotic cells with a range of CIN scores were analyzed for mitotic defects. Linear correlations were used to compare CIN by FISH to mitotic defects in tumor sections. [file crc-22-0302-s05.pdf]

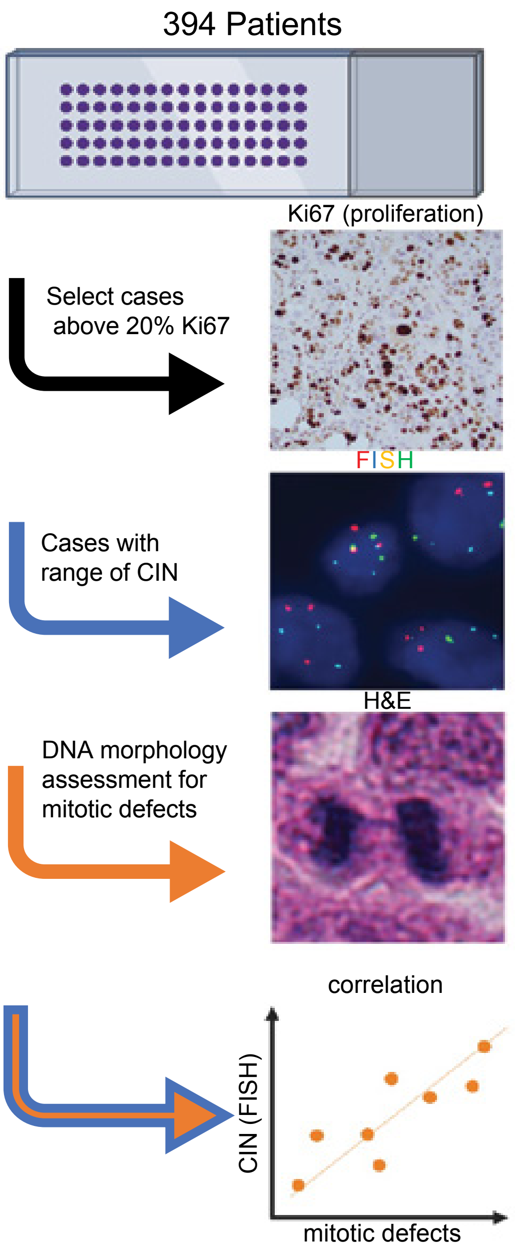

Figure S1. Pipeline to assess CIN in primary breast cancer. A. A tissue microarray containing 377 breast cancer and 17 normal breast samples was evaluated for proliferation by Ki67 and CIN based on 6 chromosome FISH (39). H&E stained sections for cases with sufficient proliferation to assess mitotic cells with a range of CIN scores were analyzed for mitotic defects. Linear correlations were used to compare CIN by FISH to mitotic defects in tumor sections.
