# Supplementary material for: Misaligned Chromosomes are a Major Source of Chromosomal Instability in Breast Cancer
Source: Cancer Res Commun. 2023 Jan 12;3(1):54–65. doi: 10.1158/2767-9764.CRC-22-0302 (PMC10035514; doi:10.1158/2767-9764.CRC-22-0302)
Supplement: Fig FS2 — Figure S2. Breast tumor subtypes show similar rates of mitotic defects. A. Triple Negative (TN) and HER2+ tumors show higher CIN as assessed by 6-chromosome interphase FISH (see Methods) than hormone (ER and/or PR) receptor positive (HR+) tumors. n = 235, 45, 46 respectively. B. Rates of mitotic defects are similar in all subtypes. [file crc-22-0302-s06.pdf]

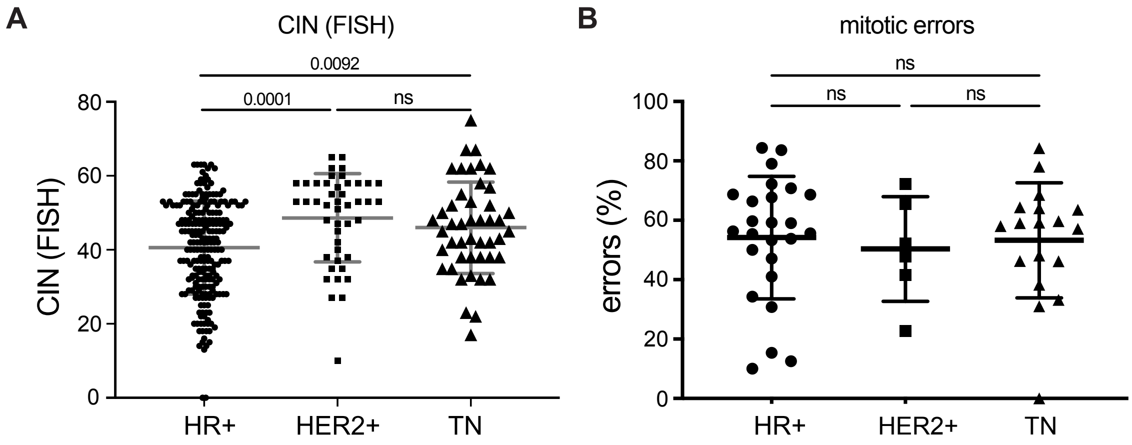

Figure S2. Breast tumor subtypes show similar rates of mitotic defects. A. Triple Negative (TN) and HER2+ tumors show higher CIN as assessed by 6-chromosome interphase FISH (see Methods) than hormone (ER and/or PR) receptor positive (HR+) tumors. n = 235, 45, 46 respectively. B. Rates of mitotic defects are similar in all subtypes.
