# Supplementary material for: Misaligned Chromosomes are a Major Source of Chromosomal Instability in Breast Cancer
Source: Cancer Res Commun. 2023 Jan 12;3(1):54–65. doi: 10.1158/2767-9764.CRC-22-0302 (PMC10035514; doi:10.1158/2767-9764.CRC-22-0302)
Supplement: Fig FS3 — Figure S3. Lagging chromosomes in breast cancer are primarily mitotic in origin. A-B. Images of lagging chromosomes lacking a centromere, which are likely to occur due to premitotic defects (A) or containing their centromere, which are likely to arise due to mitotic defects (B). C. Quantification of lagging chromosomes in 8 primary tumors showing acentric (ACA-) fragments are less frequent than lagging chromosomes containing centromeres. Data represent mean +/- SD. [file crc-22-0302-s07.pdf]

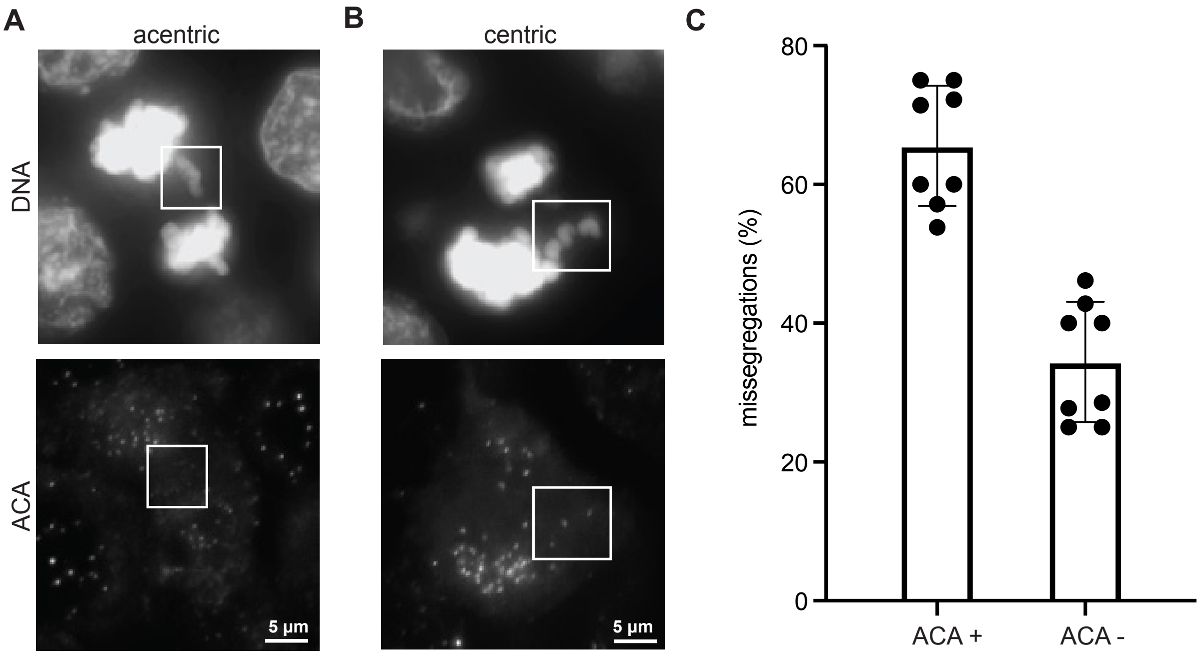

Figure S3. Lagging chromosomes in breast cancer are primarily mitotic in origin. A-B. Images of lagging chromosomes lacking a centromere, which are likely to occur due to pre-mitotic defects (A) or containing their centromere, which are likely to arise due to mitotic defects (B). C. Quantification of lagging chromosomes in 8 primary tumors showing acentric (ACA-) fragments are less frequent than lagging chromosomes containing centromeres. Data represent mean +/- SD.
