# Supplementary material for: Misaligned Chromosomes are a Major Source of Chromosomal Instability in Breast Cancer
Source: Cancer Res Commun. 2023 Jan 12;3(1):54–65. doi: 10.1158/2767-9764.CRC-22-0302 (PMC10035514; doi:10.1158/2767-9764.CRC-22-0302)
Supplement: Fig FS4 — Figure S4. Misaligned chromosomes are the predominant mitotic error in breast cancer organoids. A. Biopsies of primary breast cancer were grown in Matrigel suspension into patient-derived organoids (PDOs), which were prepared for immunofluorescence and confocal imaging. B. Representative images of mitotic cells with misaligned chromosomes observed in PDOs. C. Frequency of mitotic defects assessed in PDOs from 10 patients. P values derived from unpaired t-test. [file crc-22-0302-s08.pdf]

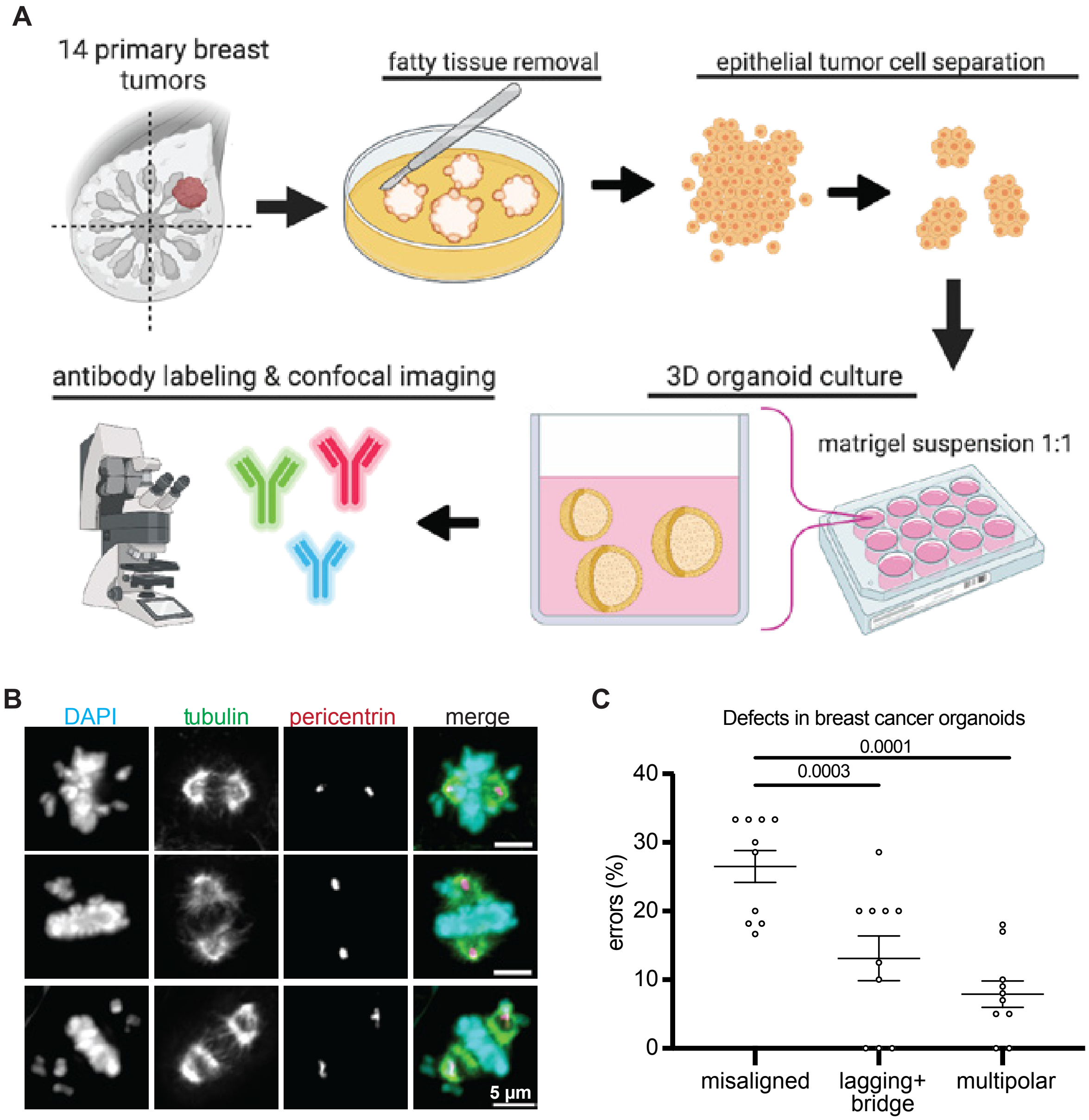

Figure S4. Misaligned chromosomes are the predominant mitotic error in breast cancer organoids. A. Biopsies of primary breast cancer were grown in Matrigel suspension into patient-derived organoids (PDOs), which were prepared for immunofluorescence and confocal imaging. B. Representative images of mitotic cells with misaligned chromosomes observed in PDOs. C. Frequency of mitotic defects assessed in PDOs from 10 patients. P values derived from unpaired t-test.
