# Supplementary material for: Misaligned Chromosomes are a Major Source of Chromosomal Instability in Breast Cancer
Source: Cancer Res Commun. 2023 Jan 12;3(1):54–65. doi: 10.1158/2767-9764.CRC-22-0302 (PMC10035514; doi:10.1158/2767-9764.CRC-22-0302)
Supplement: Fig FS5 — Figure S5. Chromosomes that are misaligned at anaphase onset are rapidly incorporated into the main DNA masses during anaphase A. A-B. Timelapse analysis of T47D (A) and Cal51 (B) breast cancer cells with endogenously labeled histone H2B and a-tubulin showing the fate of misaligned chromosomes after anaphase entry. In cells that entered anaphase with misaligned chromosomes, the majority of the misaligned chromosomes reincorporated into the main masses of segregating DNA during anaphase A (left columns). A smaller portion of misaligned chromosomes remained distinct from the segregating DNA masses and therefore visible throughout anaphase A (middle column). A small minority of misaligned chromosomes were detectable for at least 2 minutes during anaphase B (right column). n>25 cells with misaligned chromosomes in 3 biological replicates. C-D timing of anaphase and telophase in T47D (C) and Cal51 (D) cells. n>55 cells in 3 biological replicates. [file crc-22-0302-s09.pdf]

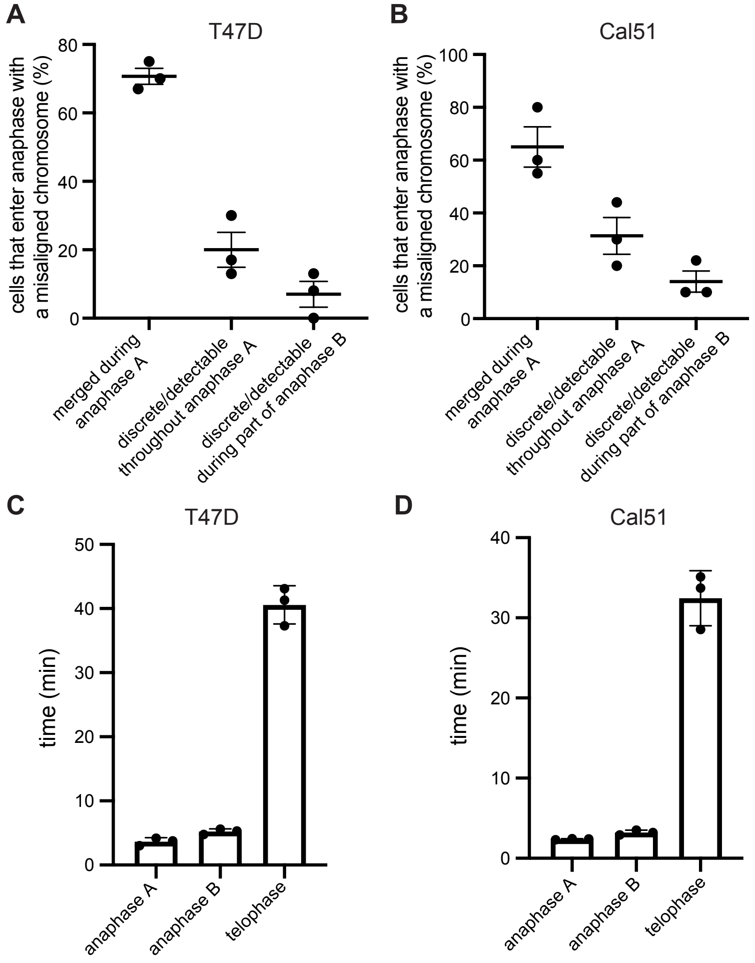

Figure S5. **Chromosomes that are misaligned at anaphase onset are rapidly incorporated into the main DNA masses during anaphase A.** A-B. Timelapse analysis of T47D (A) and Cal51 (B) breast cancer cells with endogenously labeled histone H2B and α-tubulin showing the fate of misaligned chromosomes after anaphase entry. In cells that entered anaphase with misaligned chromosomes, the majority of the misaligned chromosomes reincorporated into the main masses of segregating DNA during anaphase A (left columns). A smaller portion of misaligned chromosomes remained distinct from the segregating DNA masses and therefore visible throughout anaphase A (middle column). A small minority of misaligned chromosomes were detectable for at least 2 minutes during anaphase B (right column). n>25 cells with misaligned chromosomes in 3 biological replicates. C-D timing of anaphase and telophase in T47D (C) and Cal51 (D) cells. n>55 cells in 3 biological replicates.
