# Supplementary material for: Misaligned Chromosomes are a Major Source of Chromosomal Instability in Breast Cancer
Source: Cancer Res Commun. 2023 Jan 12;3(1):54–65. doi: 10.1158/2767-9764.CRC-22-0302 (PMC10035514; doi:10.1158/2767-9764.CRC-22-0302)
Supplement: Table TS1 — Table S1. Patient characteristics [file crc-22-0302-s10.pdf]

Table S1. Patient characteristics

Characteristic	Number	Percent
Race/Ethnicity		
Caucasian	57	96.1%
Black	3	1.7%
Asian	1	0.8%
Hispanic	0	0.6%
Unknown/Other	1	0.8%
Sex		
Female	62	100.0%
Male	0	0.0%
Age at diagnosis		
<40	7	11.3%
40-49	24	38.7%
50-59	17	27.4%
60-69	5	8.1%
70-79	7	11.3%
>=80	2	3.2%
Histology		
Ductal or ductal subtype	60	96.8%
Lobular	0	0.0%
Mammary	2	3.2%%
Phyllodes	0	0.0%
Adenoid cystic	0	0.0%
Histological Grade		
1	7	11.3%
2	12	19.5%
3	42	67.6%
Unknown	1	1.6%
Stage		
I	10	16.1%
II	43	69.4%
III	9	14.5%
Hormone Receptor Status		
ER+ and/or PR+	33	53.2%
ER/PR negative HER2+	8	12.9%
Triple negative	20	32.3
Unknown	1	1.6%
HER2 Status		
Positive	7	11.3%
Negative	50	80.6%
Unknown	5	8.1%
Regional Node Status		
Positive	32	51.6%
Negative	30	48.4%
Type of Surgery		
BCS	30	48.4%
Mastectomy	32	51.6%
No surgery	0	0.0%
Recurrence		
Yes	31	50.0%
No	31	50.0%
Vital Status		
Death due to breast cancer	22	35.5%
Death due to other cause	7	11.3%
Alive	33	53.2%
