# Supplementary material for: Misaligned Chromosomes are a Major Source of Chromosomal Instability in Breast Cancer
Source: Cancer Res Commun. 2023 Jan 12;3(1):54–65. doi: 10.1158/2767-9764.CRC-22-0302 (PMC10035514; doi:10.1158/2767-9764.CRC-22-0302)
Supplement: Table TS2 — Table S2. Patient characteristics of matched primary and metastatic cases [file crc-22-0302-s11.pdf]

Table S2. Patient characteristics of matched primary and metastatic cases

Characteristic	Number	Percent
Race/Ethnicity		
Caucasian	10	83.33%
Black	1	8.33%
Asian		0.00%
Hispanic		0.00%
Unknown/Other	1	8.33%
Sex		
Female	12	100.00%
Male	0	0.00%
Age at diagnosis		
<40	0	0.00%
40-49	5	41.67%
50-59	5	41.67%
60-69	1	8.33%
70-79	1	8.33%
>=80		0.00%
Hormone Receptor Status		
ER+ and/or PR+	8	66.67%
ER/PR negative HER2+	1	8.33%
Triple negative	3	25.00%
Unknown		0.00%
HER2 Status		
Positive	2	16.67%
Negative	10	83.33%
Unknown	0	0.00%
Recurrence		
Yes	12	100.00%
No	0	0.00%
Site		
Ovary	1	8.33%
Brain	5	41.67%
Liver	1	8.33%
Lymph Node	1	8.33%
Bone	2	16.67%
Kidney	1	8.33%
Lung	1	8.33%
