# Supplementary material for: Misaligned Chromosomes are a Major Source of Chromosomal Instability in Breast Cancer
Source: Cancer Res Commun. 2023 Jan 12;3(1):54–65. doi: 10.1158/2767-9764.CRC-22-0302 (PMC10035514; doi:10.1158/2767-9764.CRC-22-0302)
Supplement: Table TS3 — Table S3. Results from multivariable regression, related to Figure 3. [file crc-22-0302-s12.pdf]

Table S3. Results from multivariable regression, related to Figure 3.

Parameter	Estimate	P value
constant	$_{0.223}$ 0.264 $_{0.306}$	
misaligned	$_{0.017}$ 0.061 $_{0.105}$	0.008
lagging	$_{-0.004}$ 0.027 $_{0.058}$	0.088
bridge	$_{0.022}$ 0.060 $_{0.097}$	0.003
multipolar	$_{-0.111}$ -0.028 $_{0.056}$	0.510

The constant term estimates mean CIN without observable defects; other variables quantify change in mean CIN per 10 percentage point increase in the given defect. 95% confidence intervals appear as left and right subscripted values.
